# Altered cortical brain activity in end stage liver disease assessed by multi-channel near-infrared spectroscopy: Associations with delirium

**DOI:** 10.1038/s41598-017-10024-7

**Published:** 2017-08-23

**Authors:** Atsushi Yoshimura, Carrie Goodson, Jordan T. Johns, Maxwell M. Towe, Esme S. Irvine, Nada A. Rendradjaja, Laura K. Max, Andrew LaFlam, Emily C. Ledford, Julia Probert, Zoë Tieges, David H. Edwin, Alasdair M. J. MacLullich, Charles W. Hogue, Martin A. Lindquist, Ahmet Gurakar, Karin J. Neufeld, Atsushi Kamiya

**Affiliations:** 1 Department of Psychiatry and Behavioral Sciences, Johns Hopkins University School of Medicine, Baltimore, MD USA; 2 Department of Psychiatry, Shiga University of Medical Sciences, Otsu, Shiga, Japan; 3 Department of Medicine, Division of Pulmonary and Critical Care Medicine, Johns Hopkins University School of Medicine, Baltimore, MD USA; 4 Department of Biostatistics, Johns Hopkins University School of Public Health, Baltimore, MD USA; 5 Department of Anesthesiology, Johns Hopkins University School of Medicine, Baltimore, MD USA; 6 Geriatric Medicine, University of Edinburgh, Edinburgh, UK; 7 Department of Anesthesiology, Northwestern University Feinberg School of Medicine, Chicago, IL USA; 8 Department of Medicine, Gastroenterology, and Hepatology, Johns Hopkins University School of Medicine, Baltimore, MD USA

## Abstract

Delirium is a common and serious psychiatric syndrome caused by an underlying medical condition. It is associated with significant mortality and increased healthcare resource utilization. There are few biological markers of delirium, perhaps related to the etiologic heterogeneity of the syndrome. Functional near-infrared spectroscopy (fNIRS) is an optical topography system to measure changes in the concentration of oxygenated hemoglobin ([oxy-Hb]) in the cerebral cortex. We examined whether altered cortical brain activity in delirious patients with end stage liver disease (ESLD) is detected by fNIRS. We found that the [oxy-Hb] change during the verbal fluency task (VFT) was reduced in patients with ESLD compared with healthy controls (HC) in the prefrontal and bi-temporal regions. The [oxy-Hb] change during the sustained attention task (SAT) was elevated in patients with ESLD compared to HC in the prefrontal and left temporal regions. Notably, [oxy-Hb] change in the left dorsolateral prefrontal cortex during SAT showed a positive correlation with the severity of delirium. Our results suggest that [oxy-Hb] change in the prefrontal cortex during the sustained attention task measured with fNIRS might serve as a biological marker associated with delirium in ESLD patients.

## Introduction

Delirium is a common yet serious clinical syndrome characterized by fluctuating cognitive impairment. This psychiatric syndrome, produced by an underlying medical condition is primarily associated with inattention and disturbances in awareness^1^. Delirium occurs frequently throughout the healthcare system and is associated with substantial suffering and loss of dignity, longer hospital stays, institutionalization at hospital discharge, increased healthcare expenditures, increased mortality, and long-term cognitive impairment^2–8^. Although improved detection is desired, as much as 80% of delirium is currently left undiagnosed^9^. The lack of a biological marker of delirium is one hindrance to its detection.

Previous brain imaging studies using single-photon emission computed tomography (SPECT), and xenon-enhanced computed tomography (Xe-CT) have demonstrated altered cerebral perfusion in the frontal, temporal, and parietal lobes in patients with delirium^10, 11^. More recently, functional magnetic resonance imaging (fMRI) studies reported disruption of reciprocity of the dorsolateral prefrontal cortex with the posterior cingulate cortex in patients with delirium^12^. Despite the etiological complexity of delirium, these results suggest that evaluating blood flow changes in cerebral cortical regions may be useful for detecting the altered brain function underlying delirium.

Multi-channel functional near-infrared spectroscopy (fNIRS) is a method of assessing cortical brain activity by measuring regional cerebral oxygenated hemoglobin ([oxy-Hb]) and deoxygenated hemoglobin ([deoxy-Hb]) concentration changes in the cortical surface area using near-infrared light that is applied to the scalp during an activation task^13, 14^. Given that fNIRS is non-invasive, relatively fast, easily processed, and has low maintenance and measurement costs, it might be advantageous to use fNIRS as a routine tool to evaluate cortical brain activity during an activation task in high-risk patients at the bedside in clinical settings. Given the etiological complexity of delirium, studying brain activity in specific brain regions may also guide future research as we seek to understand and provide more targeted treatment for delirium.

The aim of this study is to provide a proof of concept of using fNIRS to identify changes in brain activity associated with delirium. One representative critical disease associated with delirium is end stage liver disease (ESLD); patients with acute or chronic liver failure exhibit a spectrum of neuropsychiatric abnormalities that are often referred to as “hepatic encephalopathy”^15^, which is a subtype of delirium^16^. Several imaging studies using SPECT and positron-emission tomography (PET) have demonstrated reduced cerebral oxygen consumption and blood flow in the frontal, temporal, and parietal lobes in patients with ESLD^17, 18^. Thus, we hypothesize that: 1) cortical activity during neurocognitive tasks in patients with ESLD are altered when compared to healthy controls with no liver disease (HC); and 2) delirious patients with ESLD can be differentiated from ESLD patients without delirium using fNIRS. To test these hypotheses, we measured [oxy-Hb] changes in the frontal and temporal cortex during two cognitive tasks: 1) A verbal fluency task (VFT), a test that has widely been used as an activation task for fNIRS measurement^19–22^, and 2) A sustained attention task (SAT) using the Edinburgh Delirium Test Box (EDTB) Mark 2 - a device for measuring sustained visual attention in the detection of delirium^23–25^.

## Results

### Demographic and clinical characteristics of the study groups

**Figure 1:**
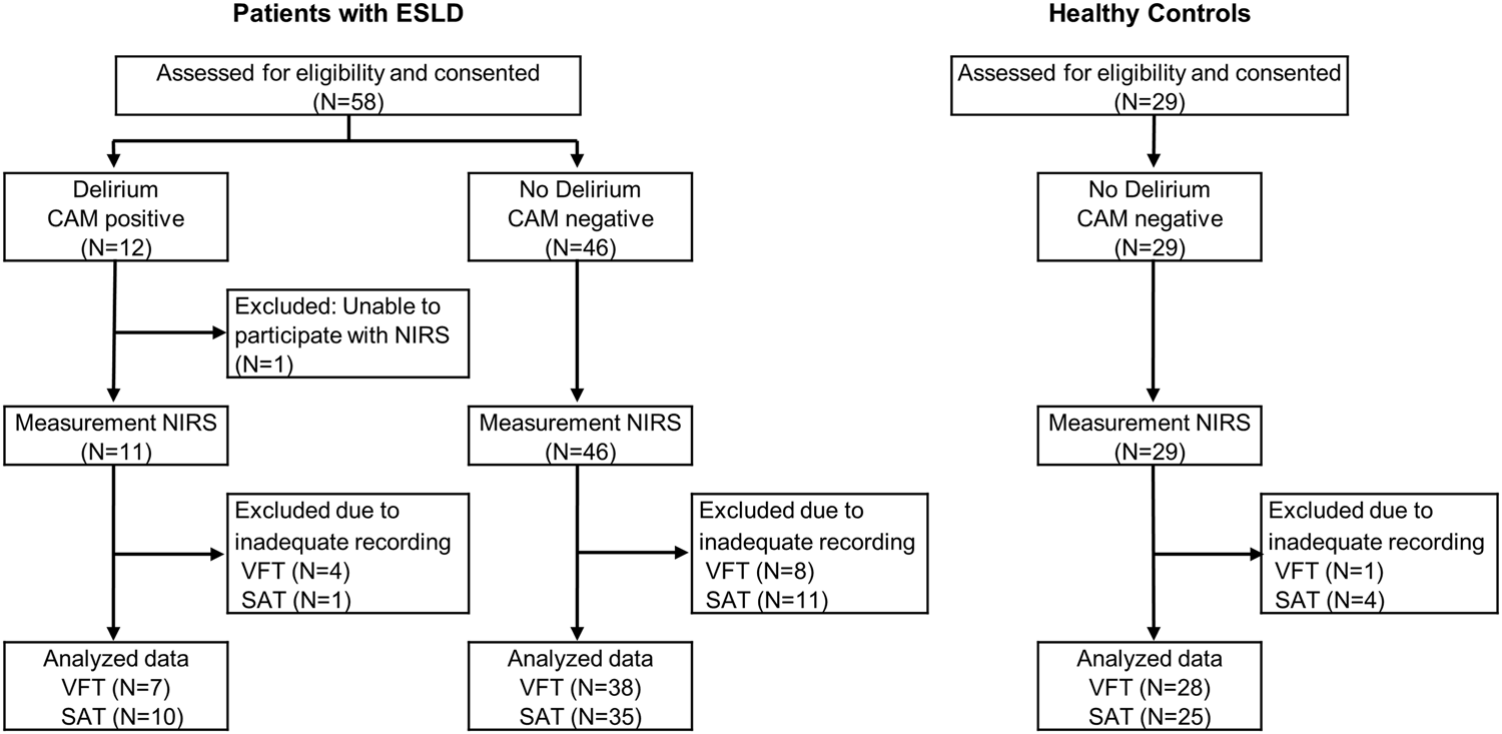
Consort diagram of participants. Near-infrared spectroscopy (NIRS) measurements were defined as inadequate when 11 channels or more contained artifacts. ESLD: End Stage Liver Disease; CAM: Confusion Assessment Method; VFT: Verbal Fluency Task; SAT: Sustained Attention Task.

Patients with end-stage liver disease (n = 58) and healthy controls (n = 29) participated in this study (Fig. 1). As shown in Table 1, apart from the distribution of race, there were no statistical differences in age, gender, or years of education between HCs and the patient group (*p* > 0.05). Patients with ESLD had higher DRS-R98 scores and poorer task performance on the VFT and SAT compared to those of HCs. Of 58 patients with ESLD, 12 (21%) were diagnosed with delirium using the CAM. There were no patients classified as Grade IV (or coma) using West Haven criteria. Distributions of the grade on West Haven Encephalopathy Criteria differ depending on CAM findings (delirium vs. no delirium in patients with ESLD (Supplementary Table 1). We found no statistical difference of use of antidepressants between ESLD patients with delirium and no delirium (Supplementary Table 1). We found no correlation of MELD scores with DRS-R98 severity scores in patients with ESLD (r = 0.155, p = 0.250) (Supplementary Fig. 1A). DRS-R98 severity scores are positively correlated with the grade of West Haven Criteria in patients with ESLD (r = 0.534, p < 0.001) (Supplementary Fig. 1B).

**Table 1:** Demographic and illness severity characteristics of study participants.

Characteristic	Patients with End Stage Liver Disease			Healthy Controls
	Delirium^a^	No Delirium^a^	Total	
N	12	46	58	29
Age Mean (SD)	59.5 (4.2)	56.5 (9)	57.1 (8.2)	53.7 (11.7)
Years of Education Mean (SD)	12.4 (2.7)	13.1 (2.5)	13.0 (2.6)	13.7 (2.9)
Male N (%)	7 (58)	31 (67)	38 (66)	21 (72)
Race N (%)				
Caucasian	12 (100)	39 (85)	51 (88)	12 (41)*
African American	0 (0)	5 (11)	5 (9)	14 (48)*
Asian/other	0 (0)	2 (4)	2 (3)	3 (10)
Admission Status N (%)				
Acute Inpatient	6 (50)	13 (28)	19 (33)	
Outpatient	6 (50)	33 (72)	39 (67)	
Medication (SD)				
Antidepressants, Imipramine-equivalent mg	51.1 (73.7)	42.3 (104.8)	44.0 (98.7)	
MELD^b^ Score Mean (SD)	15.6 (7.0)	14.9 (7.1)	15.0 (7.2)	
Charlson Comorbidity Index Mean (SD)	3.8 (1.2)	3.9 (1.5)	3.9 (1.4)	
Hemoglobin, mg/dl Mean (SD)	9.3 (2.6)	10.7 (2.6)	10.4(2.6)	
Albumin, g/dl Mean (SD)	3.2 (0.5)	3.4 (0.7)	3.3 (0.6)	
International Normalized Ratio Mean (SD)	1.4 (0.4)	1.3 (0.3)	1.3 (0.3)	
West Haven Encephalopathy Criteria^c^ N (%)				
Grade 0 – No impairment	0 (0)	11 (24)	11 (19)	
Grade I – Minor cognitive impairment	1 (8)	18 (39)	19 (33)	
Grade II – Moderate cognitive impairment	6 (50)	17 (37)	23 (39)	
Grade III – Severe cognitive impairment	5 (42)	0 (0)	5 (9)	
Grade IV - Coma	—	—	—	
DRS-R98 Severity Score^d^ Mean (SD)	12.3 (5.3)	4.3 (2.4)	6.0 (4.5)	2.3 (2.0)*
Cognitive Task Performance Mean (SD)				
Verbal fluency task^e^	8.1 (2.8)	13.8 (5.5)	12.8 (5.6)	16.9 (5.7)*
Sustained attention task^f^	0.9 (1.0)	2.5 (0.8)	2.1 (1.1)	2.8 (0.5)*

^a^As measured using the Confusion Assessment Method (CAM).

^b^MELD, Model for End-stage Liver Disease score.

^c^Severity of hepatic encephalopathy.

^d^DRS-R98, Delirium Rating-Scale Revised 98;13-item Severity subscale with possible ranges from 0 to 39.

^e^The number of words, starting with three unique letters of the alphabet correctly listed during 3 trials.

^f^The number of correct answers in three trials including practice using Edinburgh Delirium Test Box Mark 2.

*P < 0.05; P Values are calculated from the chi-squared test or a t-test between healthy controls and all patients.

### Verbal fluency task

**Figure 2:**
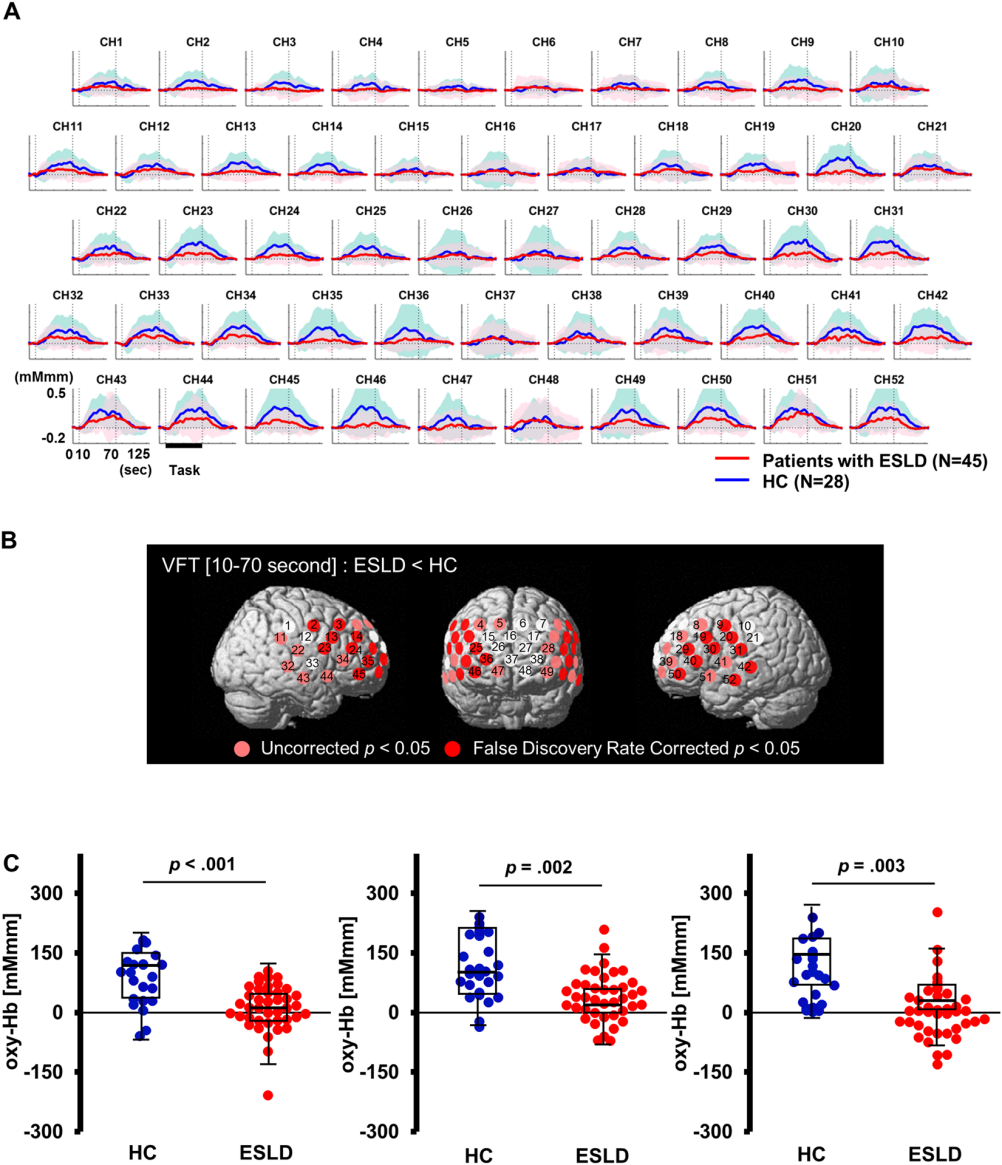
Differences between patients with end stage liver disease (ESLD) and healthy controls (HC) in the verbal fluency task (VFT). (**A**) The graphs show oxygenated hemoglobin ([oxy-Hb]) concentration change during the VFT. Standard deviations for each group are shown with thick bands. (**B**) Channels on the 3D topographic maps indicate differences in [oxy-Hb] integral value between patients with ESLD and HC (pink circles: p < 0.05 false discovery rate uncorrected, red circles: p < 0.05 false discovery rate corrected). (**C**) The graphs show differences of [oxy-Hb] integral value between HC and patients with ESLD in channel 20: left postcentral gyrus (left); channel 40: left inferior frontal gyrus (middle); and channel 46: right middle frontal gyrus (right). The upper and lower borders of the box plot marks the 75th and 25th percentiles, respectively and the middle line indicating the median value. Whiskers above and below the box indicate the highest datum still within 1.5 interquartile range (IQR) of the upper quartile, and the lowest datum still within 1.5 IQR of the lower quartile, respectively. *p < 0.05, **p < 0.01 determined by t-tests with equal variance.

The [oxy-Hb] integral value during VFT [10–70 sec] was significantly reduced in patients with ESLD compared with HC at 21 channels corresponding to the prefrontal and bi-temporal regions (channels 2, 3, 9, 13, 14, 19, 20, 23–25, 29–31, 35, 36, 40, 42, 45, 46, 50 and 52; FDR-corrected *p* < 0.05) (Fig. 2A,B). We used the mean of the [oxy-Hb] integral value during VFT [10–70 sec] among the top three channels with lowest p-value (channel 20: left postcentral gyrus, channel 40: left inferior frontal gyrus, channel 46: right middle frontal gyrus) to differentiate patients with ESLD from the controls (Fig. 2C). The resulting area under the receiver operating characteristics (ROC) curve was 0.829 [95% CI, (0.730 to 0.927)] and the optimal [oxy-Hb] integral value was 84.6 [mM·mm] from the extreme top-left point of the ROC curve. Using the threshold with 84.6 [mM·mm], the sensitivity was 91.1% (proportion of ESLD/non ESLD: 41/4) and the specificity was 64.3% (proportion of ESLD/non ESLD: 10/18), respectively. Of note, [oxy-Hb] integral value during VFT [10–70 sec] showed no significant correlation with DRS-R98 severity scores in any of the channels (Supplementary Table 2). We observed no correlation between MELD scores and [ox-Hb] integral value during VFT on any channels (Supplementary Fig. 2A). As results of group comparison among ESLD patients with delirium and non-delirium compared to HC, [oxy-Hb] integral value during VFT was significantly reduced in ESLD patients with either delirium or non-delirium, compared with HC at several channels corresponding to the prefrontal and bi-temporal regions, whereas no differences were observed between ESLD patients with and without delirium (Supplementary Fig. 3A,B and Supplementary Table 3).

### Sustained attention task

**Figure 3:**
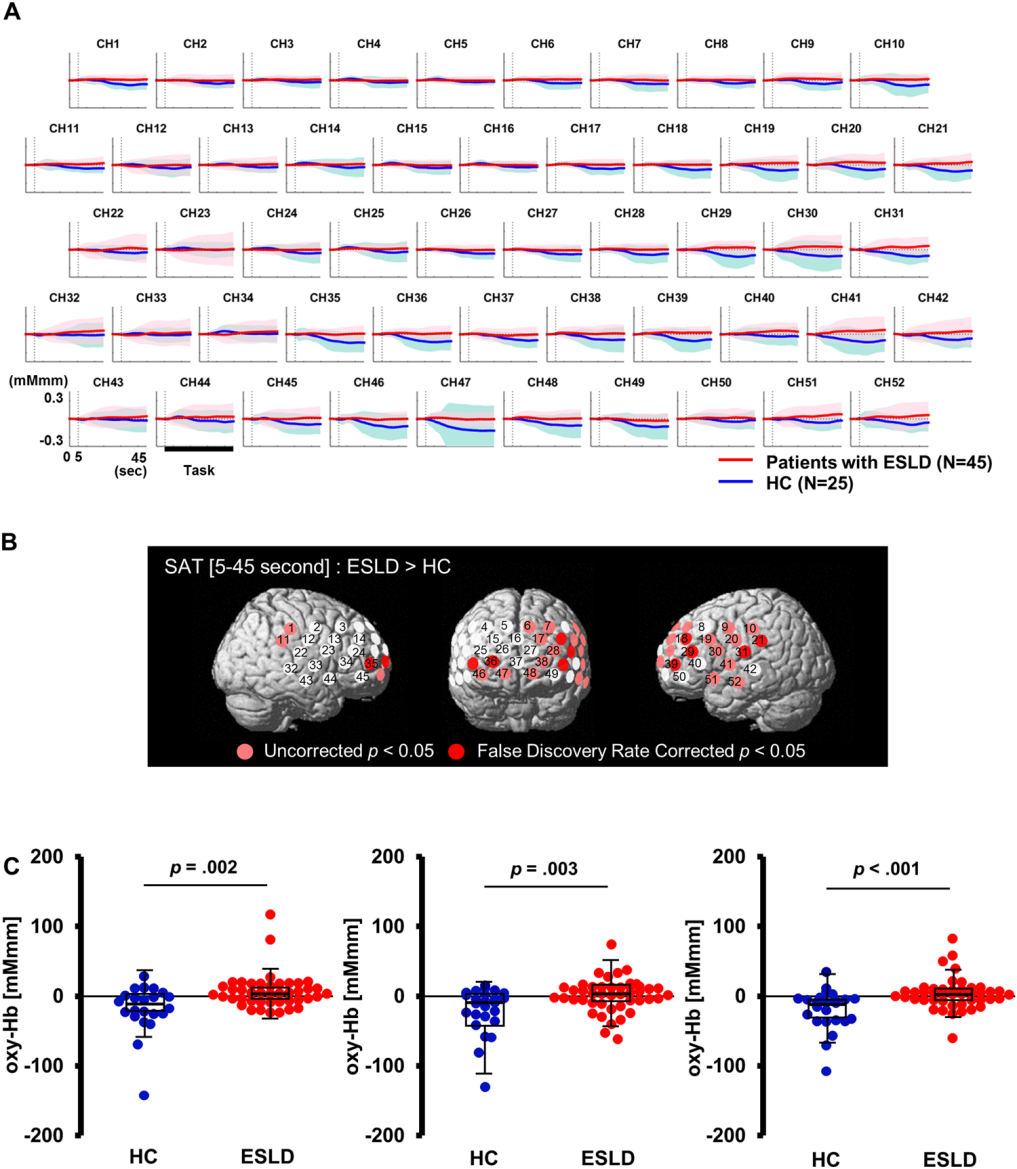
Differences between patients with end stage liver disease (ESLD) and healthy controls (HC) in the sustained attention task (SAT). (**A**) The graphs show oxygenated hemoglobin ([oxy-Hb]) concentration change during the SAT. Standard deviations for each group are shown with thick bands. (**B**) Channels on the 3D topographic maps indicate differences in [oxy-Hb] integral value between patients with ESLD and HC (pink circles: p < 0.05 false discovery rate uncorrected, red circles: p < 0.05 false discovery rate corrected). (**C**) The graphs show differences of [oxy-Hb] integral value between HC and patients with ESLD in channel29: left inferior frontal gyrus (left); channel 35: right inferior frontal gyrus (middle); and channel 36: right middle frontal gyrus (right). The upper and lower borders of the box plot marks the 75th and 25th percentiles, respectively and the middle line indicating the median value. Whiskers above and below the box indicate the highest datum still within 1.5 interquartile range (IQR) of the upper quartile, and the lowest datum still within 1.5 IQR of the lower quartile, respectively. *p < 0.05, **p < 0.01 determined by t-tests with equal variance.

**Figure 4:**
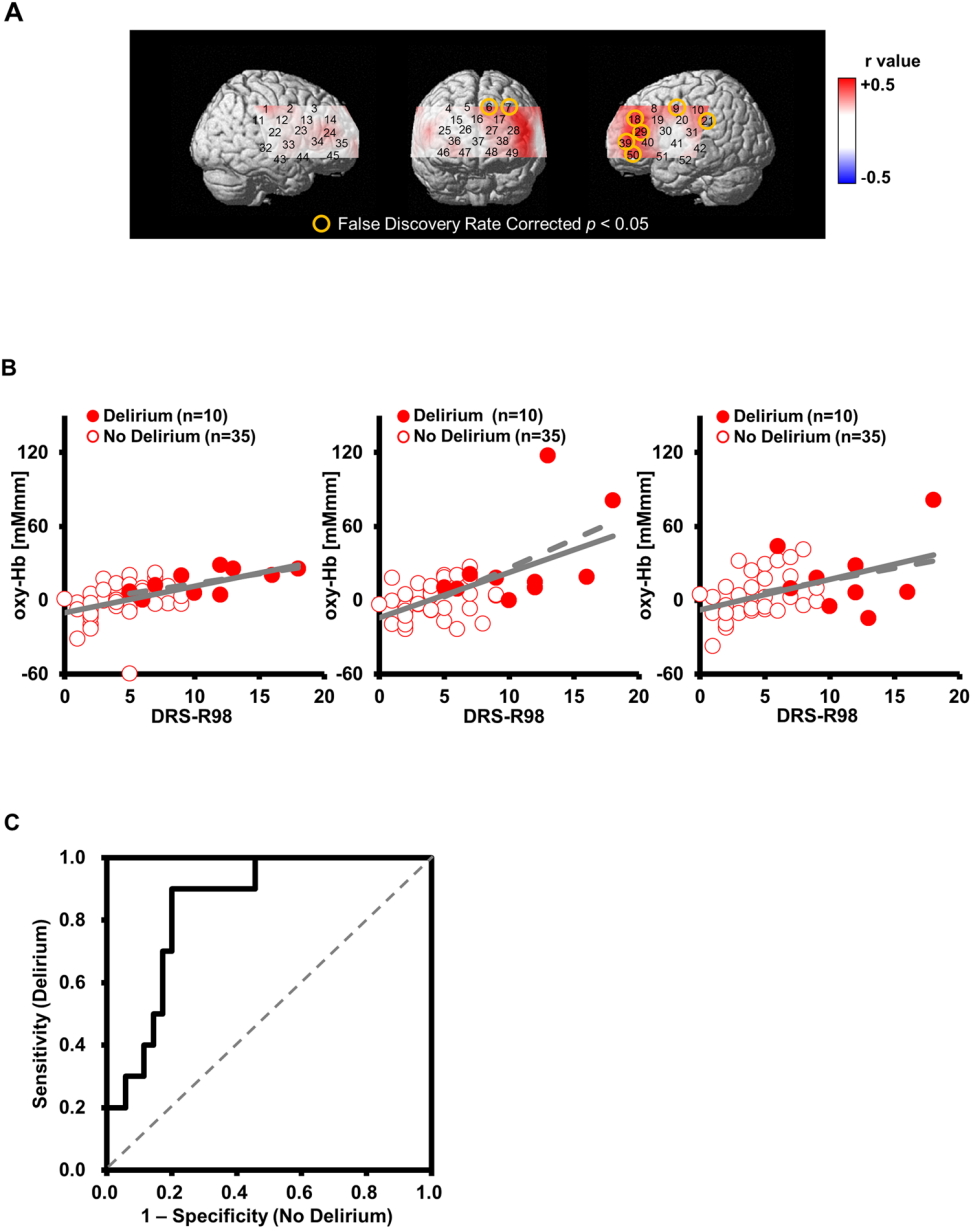
Relationship between severity of delirium measured with the Delirium Rating Scale-Revised 98 (DRS-R98) and oxygenated hemoglobin ([oxy-Hb]) integral value during the sustained attention task (SAT) in delirious and non-delirious patients with end stage liver disease (ESLD) (N = 45). (**A**) The 3D topographic maps indicate Pearson’s r values between [oxy-Hb] integral value and DRS-R98 severity scores in patients with ESLD (yellow circles: p < 0.05 false discovery rate corrected). (**B**) The scatter plots indicate correlations between [oxy-Hb] integral value and DRS-R98 severity scores in channel 18, left middle frontal gyrus (left) (Pearson’s r = 0.527, p < 0.001); channel 29, left inferior frontal gyrus (middle) (Pearson’s r = 0.600, p < 0.001); and channel 39, left inferior frontal gyrus (right) (Pearson’s r = 0.477, p = 0.001). Dotted lines are trend lines of patients with delirium. Solid lines are trend lines of all patients. (**C**) The Receiver Operating Characteristics indicate discrimination of patients with delirium from those with no delirium using mean [oxy-Hb] integral value in channel 18, 29, and 39. The area under the curve is 0.849.

The [oxy-Hb] integral value during the SAT [5–45 sec] was significantly reduced in patients with ESLD compared with HC at 7 channels corresponding to the prefrontal and left temporal region (channels 18, 21, 29, 31, 35, 36 and 39; FDR-corrected *p* < 0.05) (Fig. 3A,B). We used the mean [oxy-Hb] integral value during the SAT [5–45 sec] from the top three channels with the lowest p-value (channel 29: left inferior frontal gyrus, channel 35: right inferior frontal gyrus and channel 36: right middle frontal gyrus) to differentiate patients with ESLD from those without ESLD (Fig. 3C). The resulting area under the ROC curve was 0.749 [95% CI, (0.628 to 0.871)], and the optimal [oxy-Hb] integral value was −3.5 [mM·mm] from the extreme top-left point of the ROC curve. Using the threshold with −3.5 [mM·mm], the sensitivity was 73.3% (proportion of ESLD/non ESLD: 33/12), and the specificity was 68.0% (proportion of ESLD/non ESLD: 8/17), respectively. Importantly, in the ESLD group, [oxy-Hb] integral value during SAT [5–45 sec] showed a significant positive correlation with DRS-R98 severity scores in 8 channels (channels 6, 7, 9, 18, 21, 29, 39, 50; *r* = 0.41–0.60; FDR-corrected *p* < 0.05) that correspond to the left superior frontal gyrus, left precentral gyrus, left supramarginal gyrus, left middle frontal gyrus, and left inferior frontal gyrus (Fig. 4A). The multiple regression analysis revealed significant relationships between mean [oxy-Hb] changes in these channels during SAT and DRS-R98 scores (*R* ^2^ = 0.19–0.48, adjusted *R* ^2^ = 0.17–0.45, beta = 0.38–0.57, *p* < 0.05) after controlling for potential confounding factors, including age, gender, years of education, dose of anti-depressants (imipramine equivalent dose [mg]), MELD score, Charlson Comorbidity Index, serum hemoglobin concentration and albumin, INR, and task performance (Table 2). Performance on the SAT did not contribute significantly to [oxy-Hb] integral value in these channels. Significant relationships were found in years of education for channel 7 (left middle frontal gyrus, *R* ^2^ = 0.29, adjusted *R* ^2^ = 0.35, beta = −0.34, *p* = 0.012) and channel 29 (left inferior frontal gyrus, *R* ^2^ = 0.38, adjusted *R* ^2^ = 0.45, beta = −0.35, *p* = 0.004), and in serum albumin for channel 6 (left superior frontal gyrus, *R* ^2^ = 0.38, adjusted *R* ^2^ = 0.34, beta = 0.41, *p* = 0.002) and channel 50 (left inferior frontal gyrus, *R* ^2^ = 0.32, adjusted *R* ^2^ = 0.28, beta = 0.32, *p* = 0.019). We also observed no correlation between MELD scores and [ox-Hb] integral value during SAT on any channels (Supplementary Fig. 2B). Lastly, we used the mean [oxy-Hb] integral value during SAT [5–45 sec] among the top three channels with lowest p-value (channel 18; left middle frontal gyrus, channel 29, 39; left inferior frontal gyrus) to differentiate ESLD patients with delirium from those with no delirium (Fig. 4B). The resulting area under the ROC curve was 0.849 [95% CI, (0.732 to 0.965)] and the optimal [oxy-Hb] integral value was 6.5 [mM·mm] from the extreme top-left point of the ROC curve (Fig. 4C). Using the threshold with 6.5 [mM·mm], the sensitivity was 90.0% (proportion of delirium/no delirium: 9/1), and the specificity was 80.0% (proportion of delirium/no delirium: 7/28), respectively, for detecting CAM-positive delirious patients in the ESLD group.

**Table 2:** Multiple regression analysis based on DRS-R98 and demographic variables.

Channel number	R^2^	Adjusted R^2^	Independent variables		
			DRS-R98		Other variables
			Beta	p-Value	
Left superior frontal gyrus					
Channel 6	0.371	0.340	0.489	<0.001	Albumin: Beta = 0.412, p = 0.002
Left middle frontal gyrus					
Channel 7	0.285	0.251	0.381	0.006	Years of education: Beta = − 0.345, p = 0.012
Left postcentral gyrus					
Channel 9	0.185	0.165	0.430	0.004	
Left supramarginal gyrus					
Channel 21	0.188	0.169	0.433	0.003	
Left middle frontal gyrus					
Channel 18	0.278	0.261	0.527	<0.001	
Left inferior frontal gyrus					
Channel 29	0.480	0.454	0.569	<0.001	Years of education: Beta = −0.349, p = 0.004
Channel 39	0.228	0.209	0.477	0.001	
Channel 50	0.318	0.283	0.493	0.001	Albumin: Beta = 0.324, p = 0.019

Regression analyses were conducted after false discovery rate corrections were made at each channel.

DRS-R98, age, gender, years of education, antidepressants, model for end-stage liver disease (MELD) score, Charlson comorbidity index, serum hemoglobin concentration, serum albumin concentration, international normalized ratio (INR) and task performance were independent variables entered in the model.

## Discussion

To the best of our knowledge, this is the first study demonstrating that altered cortical activity assessed by fNIRS measurement during an activation task is associated with delirium. We evaluated ESLD patients with a subtype of delirium, hepatic encephalopathy. Altered cerebral activation and blood flow in patients with delirium has been reported in multiple studies using various brain imaging tools, such as SPECT, fMRI, and Xe-CT, suggesting that disturbances in cerebral blood flow may be important in the pathophysiology of delirium^10–12^. Although these imaging technologies have great advantages in delineating brain dysfunction in deep brain structures, several constraints, such as invasiveness, inconvenience and cost hamper their routine use in clinical settings. None of these modalities can be employed at the bedside. In contrast, fNIRS provides a non-invasive measurement of cortical function in ordinary clinical settings with relatively low cost and invasiveness. Thus, fNIRS may constitute a useful tool for assessing altered cortical brain function due to delirium at the bedside and could provide a new tool for the delirium researcher in understanding the relationship of regional cerebral activation and blood flow to the etiology of delirium.

VFT is a neuropsychological task involving multiple cognitive domains, including executive function, attention, and working memory that is widely used to study frontal and temporal lobe brain function. Abnormal cortical activity during the VFT assessed by fNIRS has been reported in multiple neuropsychiatric conditions, such as major depression, bipolar disorder, schizophrenia, and Alzheimer’s disease^19–22^. We observed significant reduction of [oxy-Hb] change during the VFT in patients with ESLD compared with HC in the prefrontal and bi-temporal regions. This is consistent with abnormalities in cerebral oxygen consumption in the frontal cortex that has been observed by fNIRS measurement during VFT and PET imaging in patients with ESLD^18, 26^. Nonetheless, we found no correlation between [oxy-Hb] changes during VFT and DRS-R98 severity scores in any brain regions in patients with ESLD. This finding suggests that VFT is not an activation task that induces measurable changes in the superficial cortex that is accessible by fNIRS in this population even though the delirious and non-delirious ESLD patient performed statistically worse than the healthy controls.

Disturbances in attention are core features of delirium of any subtype^27, 28^. In order to examine cortical function associated with sustained attention, we measured cortical hemodynamic change during the SAT, which was conducted using EDTB Mark 2, originally developed as a tool to objectively measure attention in delirium assessment^23, 25^. Considering that patients with severe delirium would be expected in clinical settings, we attempted to lighten the task load by modifying the original program of EDTB Mark2, which consists of 16 trials with an overall duration of 8 minutes, to a version with only 3 trials in 4 minutes including resting periods. This also was done to maximize patient motivation, since a lack of subject motivation could be falsely attributed to delirium in this task and confound our results. We found a significant increase of [oxy-Hb] change in the prefrontal and bi-temporal regions during the SAT in patients with ESLD compared to the HC group. Of note, DRS-R98 severity scores showed a strong positive correlation with [oxy-Hb] change during SAT in the left dorsolateral prefrontal cortex, which is a critical brain region for regulating attention^29^. Functional abnormalities in the left dorsolateral prefrontal cortex have also been reported in patients with delirium^12^. While seemingly contradictory to previous findings of decreased cerebral blood flow in delirium vs resolved delirium^10, 11^, it is important to note that our study demonstrated task-related changes in cerebral blood flow rather than resting cerebral blood flow. Our results suggest that cortical activity during SAT in certain channels corresponding to attention-related brain regions is altered in hepatic encephalopathy (a subtype of delirium). Taken together, fNIRS, as a noninvasive portable method for monitoring changes in cerebral blood flow, may be useful for assessing delirium in patients with ESLD and understanding the underlying pathophysiology of the syndrome.

Slooter and colleagues have recently reported that endo-phenotypes assessed by eye-closed EEG recording may be a useful biomarker for detecting delirium in patients undergoing cardiothoracic surgery^30^. While the task requirement during fNIRS measurement in this exploratory study makes it an active task instead of a passive measure (as in EEG), it provides a unique opportunity to detect real-time altered regional brain function associated with a sustained attention. Furthermore, fNIRS is a portable method easily and quickly applied, making it a convenient adjunct to bedside testing for delirium, which currently is underdiagnosed in many clinical settings^31, 32^. Given that inattention is a core feature of delirium of any subtype, this method could be used in populations of delirious patients due to causes other than end stage liver disease to test its suitability as a biological marker for diagnosis of delirium.

There are several limitations of this study. We recruited only patients who could cooperate with activation tasks and NIRS scanning. Thus, selection bias should be considered when generalizing our findings to patients with severe physical or delirious conditions who are not capable of undergoing fNIRS measurement tasks. While the false discovery rate correction was applied to minimize the number of false positive findings, our results require prospective verification using a predetermined group of fNIRS channels in the left dorsolateral prefrontal cortex, to test the reproducibility of these exploratory findings, provide a more concise measurement protocol and make this method more realistic in a clinical setting. Given that the number of patients with delirium was relatively small at n = 10, the statistical power for assessing diagnostic accuracy for delirium is limited. The modest sample size also precluded our ability to evaluate the effect of depression or anti-depressants on our results. Moreover, because DRS-R98 scores may be confounded by existing symptoms besides delirium (e.g., mild cognitive impairment), the prospective evaluation of delirium and cognitive function in individual subjects is needed. Delirium is a heterogeneous syndrome condition with multiple etiologies^7, 8, 33^. Thus, the utility of measurement of altered cortical activity during SAT by fNIRS for diagnosis of delirium should be validated in delirious patients with non-hepatic disease conditions. Nevertheless, this line of research may contribute to developing biological markers of delirium, an underdiagnosed syndrome of major prognostic significance in the clinical setting.

Because research in the field of delirium has been hampered by the heterogeneous etiologies and unclear mechanisms, having a noninvasive method for assessing brain function associated with delirium at the bedside could lead to significant progress in this field toward improved detection and understanding of the mechanisms associated with the syndrome. Our study revealed potentially important abnormalities in brain activity mediated by the SAT and the VFT in patients with ESLD compared with HC. In addition, SAT-mediated [oxy-Hb] changes in specific brain regions were associated with delirium diagnosis and DRS-R98 severity scores in the patient group. Considering its non-invasive nature, relatively low cost, and its portability, these findings suggest that fNIRS monitoring may be a useful biological marker for evaluating and detecting functional abnormalities associated with the pathophysiology of delirium in patients with liver disease. Future studies are needed to determine if cerebral perfusion, evaluated with fNIRS, is an important mechanism in patients with non-hepatic delirium as well.

## Methods

### Participants

Patients with ESLD were recruited from the Johns Hopkins Liver Transplant Clinic. Healthy controls (HC) were recruited from several sources including the relatives of the patients in the Liver Transplant Clinic, from community-based advertising through the internet, and flyers posted at hospital and university campuses. Exclusion criteria were: 1) inability to speak and/or understand English; 2) severe hearing or visual impairment; 3) previous stroke or head trauma; 4) use of antipsychotics; 5) diagnosis of schizophrenia or bipolar disorder. All participants were recruited from November 2014 to June 2016. The delirium assessment and fNIRS measurement were sequentially conducted.

### Ethics Statement

The study protocol was approved by the Johns Hopkins IRB (IRB00036271). In accordance with the Declaration of Helsinki, all participants and their legally authorized representatives gave written informed consent.

### Data collection

Baseline demographics, laboratory data and baseline health variables were collected from all participants. Liver function in patients was documented using the Model for End-Stage Liver Disease (MELD) score, which is employed for survival prediction in patients with liver cirrhosis^34^. Additional clinical information, including dose of antidepressants (imipramine equivalent dose [mg]), Charlson Comorbidity Index^35^, serum hemoglobin concentration (mg/dl), serum albumin (g/dl), and International Normalized Ratio (INR) were obtained from individual medical records. The severity of cognitive dysfunction associated with ESLD (also called “hepatic encephalopathy”) was assigned by research staff using the West Haven criteria – a scale which assigns cognitive impairment from none (Grade 0), minor, moderate or severe (Grades I–III, respectively) and coma (Grade IV)^15^ after testing the patient and interviewing a patient informant about the individual’s baseline.

### Delirium training and assessment

All participants underwent standardized cognitive screening and a clinical interview by a trained research assistant who rated the long form of the Confusion Assessment Method algorithm (CAM)^36^ and the Delirium Rating Scale-Revised 98 (DRS-R98), a 13-item scale with a maximum severity score of 39^37^. While the gold standard diagnostic criteria for delirium are from the Diagnostic and Statistical Manual (DSM)^1^, we used CAM and DRS-R98 ratings because of the superior correlation in delirium diagnosis between DSM-IV and either CAM or DRS-R98 compared to correlation between DSM-IV and DSM-V^38^. Liberal vs conservative application of DSM-V criteria alone affects delirium diagnosis^39^ and a more liberal application is recommended^40^. CAM and DRS-R98 were rated based on the patient’s performance on a standardized cognitive screening battery and history about the patient’s baseline obtained from collateral sources.

Research assistants underwent extensive training in delirium assessment before the start of study under the supervision and training of a Board Certified Psychiatrist (K.J.N). This included interview and independent rating of at least 10 non-study subjects until 5 consecutive assessments were 100% concordant for CAM algorithm assignment and DRS scoring when independently observed and co-rated by K.J.N. Quality assessment training continued throughout the study with research assessors being observed and their assessments curated by the delirium measurement expert (K.J.N) and each other. Agreement was high with kappas ranging from 0.70 to 0.81^41^. All cognitive and delirium assessments were conducted within one hour of the fNIRS measurement; trained research assistants performing the delirium assessments (M.M.T., A.L., L.K.M., E.S.I., N.A.R., J.P., E.C.L.) were blind to the results of the fNIRS, which was performed separately by a research fellow (A.Y).

### Verbal fluency task

A modified version of the VFT was employed as a cognitive activation task during fNIRS measurement. Participants were instructed to relax in a chair and keep their eyes open during the task. Participants performed a VFT consisting of a 30 second (s) pre-task baseline period, a 60-s task period, and a 70-s post-task baseline period^19^. During the 60-s task period, participants were instructed to verbally list as many words as possible that begin with a given letter of the alphabet without using repetitions and proper nouns. The task period was split into three; participants were required to generate words using one of three letters during each of these sub-periods (1^st^ period: letters “f”, “c”, or “e”; 2^nd^: “a”, “g”, or “d”; 3^rd^: “s” “r” or “o”). Letters were changed every 20 s to prevent participants from being silent. In the 30-s pre-task and 70-s post-task periods, participants were instructed to continuously repeat the first five letters of the alphabet (i.e., “a, b, c, d, e, a, b, c, d, e” and so on). The total number of correct words generated during the 60 s VFT period was used as the measure of task performance. To minimize vocalization effects during the task, activation during pre- and post-task period was subtracted from the fNIRS measurement during the task period (Supplementary Fig. 4A and Appendix 1 in the data supplement).

### Sustained attention task

The EDTB Mark 2, a computerized neuropsychological testing device, was used as sustained attention task (SAT) during fNIRS measurement. The test has been validated as a method of detecting delirium and discriminating it from dementia^24^. The EDTB Mark 2 features two large illuminable buttons (5 cm in diameter) and a 7 × 7 array of additional light-emitting diodes to display distracting stimuli (Supplementary Fig. 4B). Participants were asked to count the number of times the buttons illuminated, excluding distractor lights that are flashed over the course of the trial. Participants then verbally reported that number upon completing each session. The test originally consisted of five graded tasks with progressively more difficult attentional demands. In this study, we adapted Grade 5, which consists of three trials. We excluded the data of fNIRS measurement in the first trial used as a practice session, and we analyzed the data from the second and third trials according to the time course of the study design (Supplementary Fig. 4C and Appendix 2 in the data supplement). Each trial consisted of 7 to 13 illuminations of the buttons. Illuminations lasted 1 s with an inter-trial interval ranging between 1 and 4 s. Task performance was rated as binary, by assigning a score of 1 to each correct answer, including the practice trial, amounting to a summed total score ranging from 0 to 3.

### fNIRS measurement

The [oxy-Hb] and [deoxy-Hb] concentration changes were assessed using 52-channel fNIRS (ETG-4000; Hitachi, Ltd., Tokyo, Japan), which measures the absorption of two wavelengths of near-infrared light (695 and 830 nm) (Supplementary Fig. 4D). The [oxy-Hb] and [deoxy-Hb] were calculated according to the modified Beer-Lambert Law^42^. The distance between the pair of emission and detection probes was set at 30 mm. The fNIRS measured hemoglobin ([Hb]) concentration change at the midpoints between the probes at depths of 20–30 mm from the scalp^43^. Each midpoint between two probes is considered one channel and this device includes 52 channels arranged in a 6 × 30 cm area. The NIRS probes were placed on a participant’s frontal and temporal cortical region with the mid-column of the probe located over Fpz in accordance with the international 10/20 system used in electroencephalography^44^. This arrangement of probes measured [Hb] concentration change from the bilateral prefrontal cortical areas (i.e., frontopolar [FP; approximately corresponding to Brodmann’s area (BA) 10], dorsolateral [DL; BA 9, 46], ventrolateral [VL; BA 44, 45, 47]), and superior temporal cortical surface regions) is an approach supported by a multi-subject study of anatomical craniocerebral correction using the international 10/20 system^44^. Cortical regions corresponding to each channel were estimated using a virtual registration method^45^. The absorption of near-infrared light was measured with a time resolution of 0.1 s. The data during VFT was analyzed using the “integral mode”: the pre-task baseline was determined between 20 s and 30 s of the pre-task period, and the post-task baseline was determined between 140 s and 145 s of the post-task period. Linear fitting was applied to the data between these two baselines as previously described^46^. The data from the SAT were analyzed with the pre-task baseline, which was determined as the mean across the last 5 s of the 40 s resting period. Linear fitting (degree = 0) was applied to the pre-task baselines. The moving average method (5 s moving average window) was used to exclude short-term motion artifacts in data analysis. Automatic artifact-rejection procedures were also carried out to exclude artifact waveforms in accordance with several criteria with each task (see Appendix 2 and 3 in the data supplement). When fewer than 42 channels (or 80% of all channels) met these criteria the results were excluded from the data analysis (Fig. 1). The success rate of the fNIRS procedure in ESLD patients was 98.3% (57/58) (Fig. 1). One patient was not applicable for fNIRS measurement because of the hyperactive type of delirium. The error rate for the body-movement artifacts of fNIRS data on ESLD patients was 21% (12/58). We needed approximately five minutes for setting up fNIRS measurement including mounting fNIRS probes on participant’s head and practice sessions with each active task. Approximate eight minutes was needed to complete fNIRS measurement with each activation task.

### Statistical analysis

The [oxy-Hb] changes during an activation task were converted into an integral value, which represents the total hemodynamic response across time (or area under the curve). The [oxy-Hb] integral value during the VFT and SAT were compared between patients with ESLD and HC for each channel using two-sample t-tests with equal variance with two-sided alternative hypotheses. Pearson’s correlation coefficients were calculated for each channel to assess the relationship between [oxy-Hb] integral value and DRS-R98 severity scores in the patient group. Because we performed 52 t-tests, we applied the false discovery rate (FDR) correction to correct for multiple comparisons. We specified an FDR value of q = 0.05 to ensure a false-positive rate no more than 5% on average^47^. For the channels with significant correlation between [oxy-Hb] integral value and DRS-R98 scores, we performed stepwise multiple linear regression analysis (entry: *p* = 0.05; and removal: *p* = 0.10) to evaluate the relationship between [oxy-Hb] integral values and various demographic characteristics, including gender, age, years of education, biological MELD score, antidepressants, Charlson Comorbidity Index, peripheral hemoglobin concentration, serum albumin, international normalized ratio, DRS-R98 scores, and task performance. All statistical analyses were performed using SPSS 23 (IBM Inc., Armonk, NY, USA) and MATLAB R2015a (MathWorks Inc., Natick, MA, USA) software.

## Electronic supplementary material

Supplementary Informatrion
