# Supplementary material for: Altered cortical brain activity in end stage liver disease assessed by multi-channel near-infrared spectroscopy: Associations with delirium
Source: Sci Rep. 2017 Aug 23;7:9258. doi: 10.1038/s41598-017-10024-7 (PMC5569013; doi:10.1038/s41598-017-10024-7)
Supplement: Supplementary file 1 — Supplementary Informatrion [file 41598_2017_10024_MOESM1_ESM.pdf]

Altered cortical brain activity in end stage liver disease assessed by multi-channel near-infrared spectroscopy: Associations with delirium

Atsushi Yoshimura, M.D. Ph.D.[1,2], Carrie Goodson, M.D. M.H.S.[3], Jordan T Johns[4], Maxwell M. Towe, B.A.[1], Esme S Irvine, B.A.[1], Nada A Rendradjaja, B.A.[1], Laura K Max[5], Andrew LaFlam[5], Emily C Ledford[5], Julia Probert[5], Zoë Tieges, Ph.D.[6], David H Edwin, Ph.D.[1], Alasdair MJ MacLullich, M.B., Ch.B., M.R.C.P., Ph.D.[6], Charles W Hogue, M.D.[5, 7], Martin A. Lindquist, Ph.D.[4], Ahmet Gurakar, M.D.[8], Karin J Neufeld, M.D. M.P.H.[1,9], Atsushi Kamiya, M.D. Ph.D.[1,9]

[1]Department of Psychiatry and Behavioral Sciences, Johns Hopkins University School of Medicine, Baltimore, MD, USA; [2]Department of Psychiatry, Shiga University of Medical Sciences, Otsu, Shiga, Japan; [3]Department of Medicine, Division of Pulmonary and Critical Care Medicine, Johns Hopkins University School of Medicine, Baltimore, MD, USA; [4]Department of Biostatistics, Johns Hopkins University School of Public Health, Baltimore, MD, USA; [5]Department of Anesthesiology, Johns Hopkins University School of Medicine, Baltimore, MD, USA; [6]Geriatric Medicine, University of Edinburgh, Edinburgh, UK; [7]Department of Anesthesiology, Northwestern University Feinberg School of Medicine, Chicago, IL, USA; [8]Department of Medicine, Gastroenterology, and Hepatology, Johns Hopkins University School of Medicine, Baltimore, MD, USA; [9]Drs. Neufeld and Kamiya are co-corresponding authors and have contributed equally as senior authors on this manuscript.

Address correspondence to Atsushi Kamiya. E-mail: akamiya1@jhmi.edu and Karin J Neufeld. E-mail: kneufel2@jhmi.edu.

Supplementary materials

Appendix. 1: Verbal fluency task

The data were analyzed using the “integral mode”: the pre-task baseline was determined between 20 s and 30 s of the pre-task period, and the post-task baseline was determined between 140 s and 145 s of the post-task period. Linear fitting (degree=1) was applied to the data between these two baselines. The moving average method (5 s moving average window) was used to exclude short-term motion artifacts to analyze the data. We also used a computer program to reject a channel when artifact waveforms were visible(Takizawa R et al. 2014).

High-frequency noise is detected using standard deviation (SD). Thus, we calculated the SD value of four blocks (20–35 [s], 35–50 [s], 50–65 [s], and 65–80 [s]) from oxy-hemoglobin (oxy-Hb) [x_{oxy} (t)], deoxy-hemoglobin (deoxy-Hb) [x_{deoxy} (t)], and total-hemoglobin (Hb) [x_{Total} (t): total-Hb = oxy-Hb + deoxy-Hb], using Equation (1):

$$SD_{hb} = \sqrt{\frac{1}{n-1}\sum_{t=1}^{N}(x_{hb}(t) - \bar{x}_{hb})^2} \; , \qquad (1)$$

Where n is the number of measurement points.

In such cases, if the channel’s SD_{Oxy} value in each block is over SD_{Total} ×4, and its SD_{Deoxy} value in each block is over SD_{Total} ×4, the channel is determined as an artifact channel.

Low-frequency noise is detected using the low-frequency value (LF) and the correlation value (r). The LF is given by Equation (2):

$$LF = abs\left(1 - \frac{\sqrt{\frac{1}{n-1}\sum_{t=1}^{N}(x_{Deoxy}(t) - \bar{x}_{Deoxy})^2}}{\sqrt{\frac{1}{n-1}\sum_{t=1}^{N}(x_{Oxy}(t) - \bar{x}_{Oxy})^2}}\right), \qquad (2)$$

where N is the number of measurement points. LF was n = 1251.

We calculated the correlation value between oxy-Hb and deoxy-Hb at each time point.

$$r = \frac{\sum_{t=1}^{N} x_{Oxy}(t) x_{Deoxy}(t)}{\sqrt{\sum_{t=1}^{N} x_{Oxy}(t)^2}\sqrt{\sum_{t=1}^{N} x_{Deoxy}(t)^2}}, \qquad (3)$$

Where n is the number of measurement points. The value of r was n = 1251. We also determined that an artifact channel(s) had an LF < 0.3 and an r value < -0.9.

The frequency with no signal had no changes in the concentrations of oxy-Hb or deoxy-Hb in any of the measurement time points. Therefore, the channels in which the SD values of all the measurement points were 0 were determined to be artifact channels.

The body-movement artifacts showed sharp changes. Channels that had body-movement artifacts with oxy-Hb and total-Hb changes over 0.25 [mM·mm] in 20 successive samples (during 2 [s]) were determined as artifact channels.

Appendix. 2: Sustained attention task using Edinburg delirium test box Mark 2

The data were analyzed using the “integral mode”: the pre-task baseline was determined between 25 s

and 30 s of the resting period. Linear fitting (degree=0) was applied to the pre-task baselines. The moving average method (5 s moving average window) was used to exclude short-term motion artifacts in the data analysis. We also used a computer program (Appendix. 1) to reject a channel when artifact waveforms were visible.

Appendix. 3: Method of automatic rejection of artifacts

To obtain reliable data, we developed an algorithm to automatically detect frequency channels that indicate noise artifacts and body-movement artifacts.

There are two kinds of noise artifacts (low signal-to-noise ratio and no signal), and those body-movement artifacts show sharp signal changes compared to those of normal hemodynamics.

Low signal-to-noise ratio is detected using standard deviation (SD). Thus, we calculated the SD value during the pre-task period (0-5 s) from oxy-hemoglobin (oxy-Hb) [x_{oxy} *(t)*] and deoxy-hemoglobin (deoxy-Hb) [x_{deoxy} *(t)*] values, using Equation (1):

$$SD_{hb} = \sqrt{\frac{1}{n-1}\sum_{t=1}^{N}(x_{hb}(t) - \bar{x}_{hb})^2} \; , \qquad (1)$$

where n is the number of measurement points. In such cases, if the SD_{Oxy} and SD_{Deoxy} values of the channel were over 0.04, then the channel was classified as an artifact channel.

The frequency with no signal exhibited no changes in [oxy-Hb] or [deoxy-Hb] at any of the measurement time points. Therefore, the channels in which the *SD* values of all the measurement points were 0 were classified as artifact channels.

The body-movement artifacts showed sharp changes. Therefore, channels that exhibited body-movement artifacts with [oxy-Hb] and [total-Hb] changes over 0.05 [mMmm] in 20 successive samples (during 2 s) were classified as artifact channels.

References

Takizawa R, Fukuda M, Kawasaki S, Kasai K, Mimura M, Pu S, Noda T, Niwa S, Okazaki Y. 2014. Neuroimaging-aided differential diagnosis of the depressive state. NeuroImage 85 Pt 1:498-507.

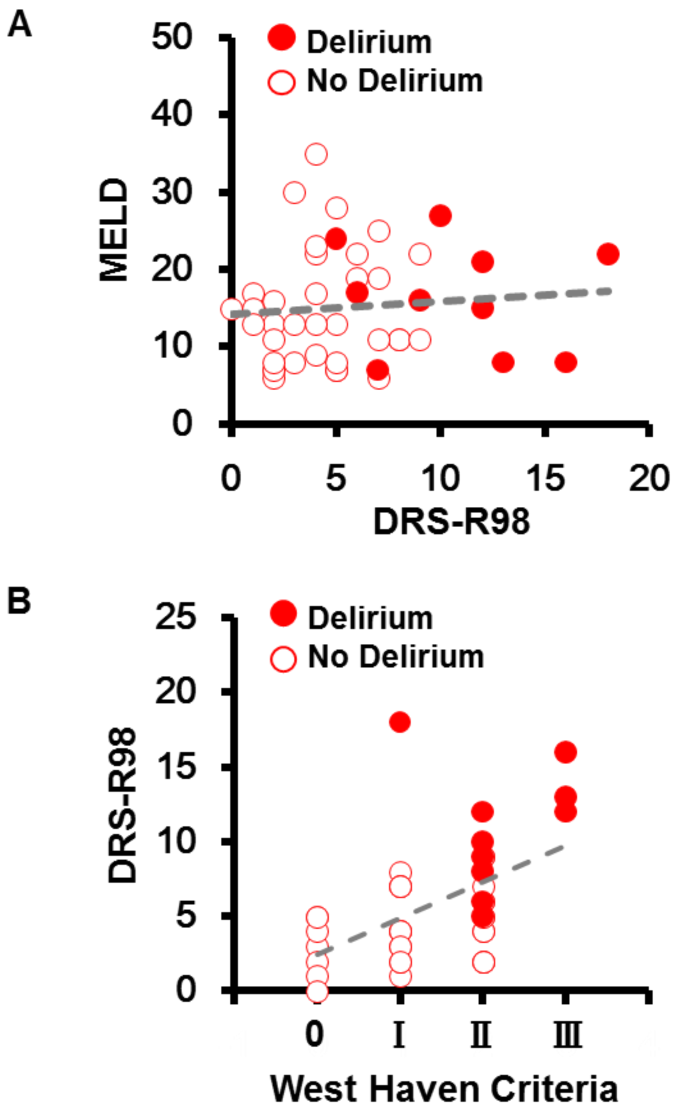

Figure Supplementary

Fig. 1. Relationship between severity of delirium measured with the Delirium Rating Scale-Revised 98 (DRS-R98) and the model for end-stage liver disease (MELD) score, or the West Haven criteria in patients with end stage liver disease (ESLD) (N=45). (A) The scatter plots indicate correlations between MELD score and DRS-R98 severity scores (Pearson's r = 0.088, p = .516). Dotted lines are trend lines of all patients. (B) The scatter plots indicate correlations between grade of West Haven Criteria and DRS-R98 severity scores (Pearson's r = 0.579, p < .001). Dotted lines are trend lines of all patients.

A

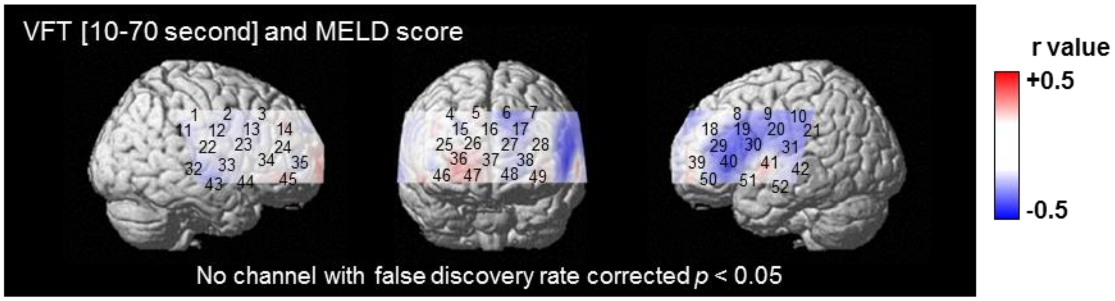

B

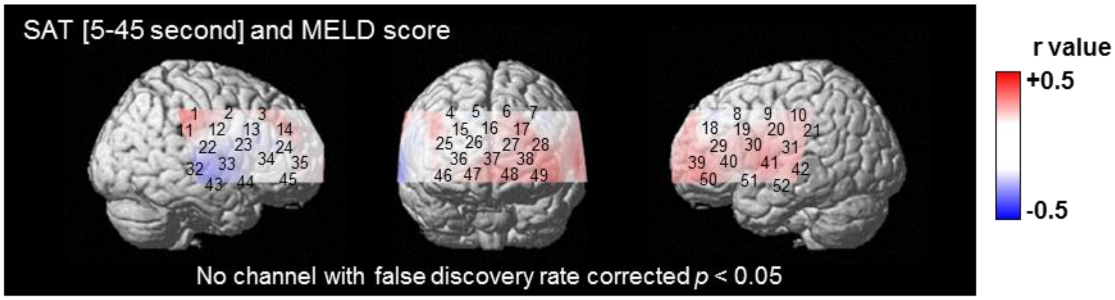

Figure Supplementary

Fig. 2. Relationship between severity of liver function evaluated with the model for end-stage liver disease (MELD) score and oxygenated hemoglobin ([oxy-Hb]) integral value during each task in delirious and non-delirious patients with end stage liver disease (ESLD) (N=45). (A) The 3D topographic maps indicate Pearson's r values between [oxy-Hb] integral value during the verbal fluency task (VFT) and MELD scores in patients with ESLD. (B) The 3D topographic maps indicate Pearson's r values between [oxy-Hb] integral value during the sustained attention task (SAT) and MELD scores in patients with ESLD.

A

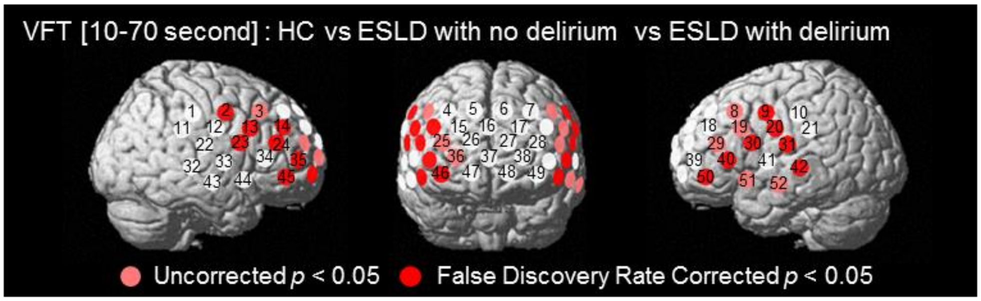

B

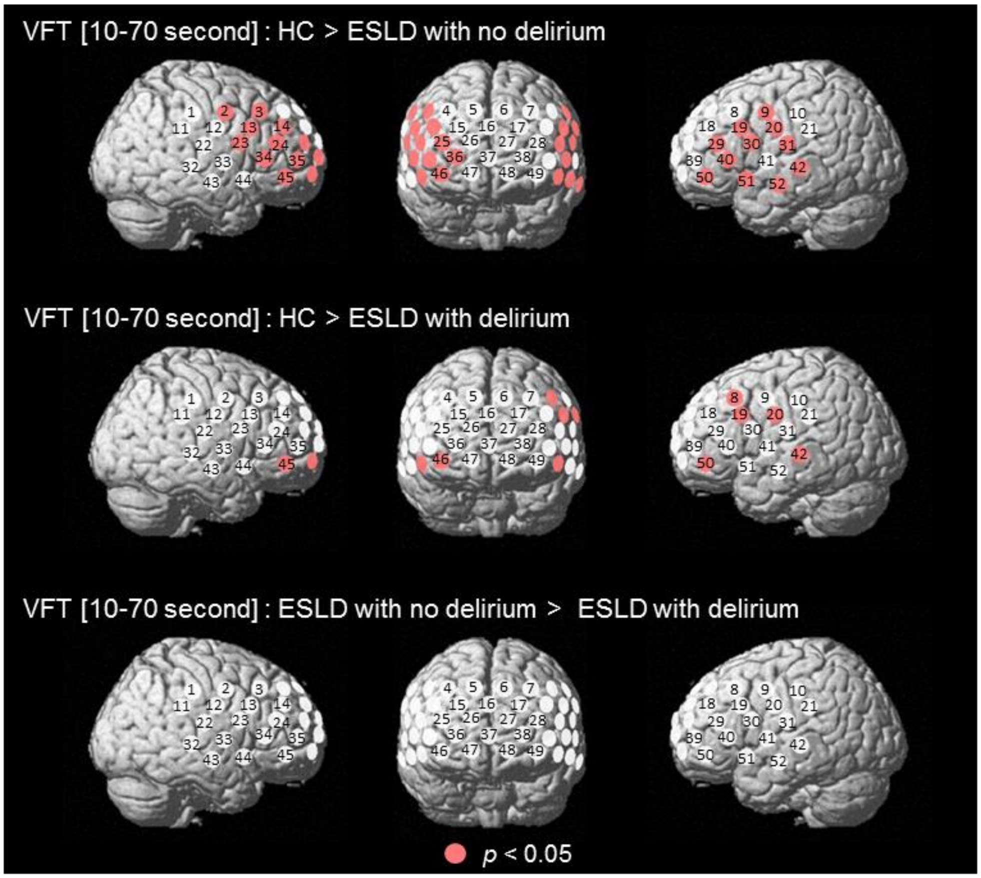

Figure Supplementary

Fig. 3. Differences among healthy controls (HC), patients with end stage liver disease (ESLD) with no delirium and ESLD with delirium in the verbal fluency task (VFT). (A) Channels on the 3D topographic maps indicate differences in [oxy-Hb] integral value among HC, ESLD with no delirium and ESLD with delirium. Differences among 3 groups were tested by one-way analysis of variance (ANOVA) for each

channel (pink circles: $p<0.05$ false discovery rate uncorrected, red circles: $p<0.05$ false discovery rate corrected). (B) The channels indicating differences in [oxy-Hb] integral value by post hoc Tukey–Welsch tests (pink circles: $p<0.05$).

A.

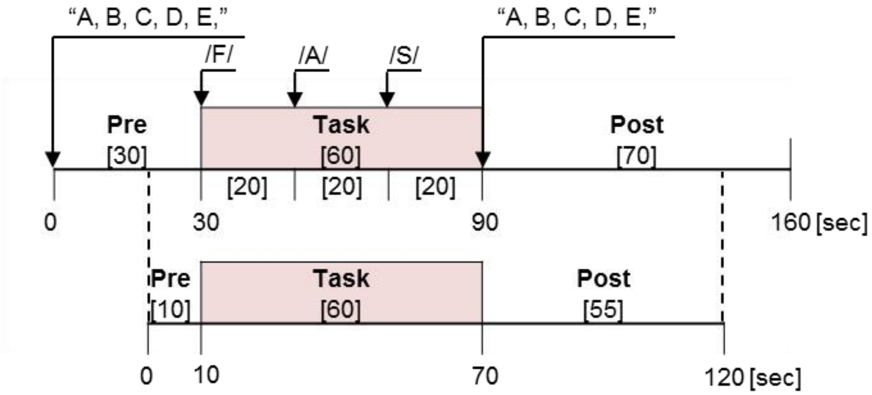

"A, B, C, D, E,"
/F/
/A/
/S/
"A, B, C, D, E,"
Pre
[30]
Task
[60]
Post
[70]
[20]
[20]
[20]
0
30
90
160 [sec]
Pre
[10]
Task
[60]
Post
[55]
0
10
70
120 [sec]

B.

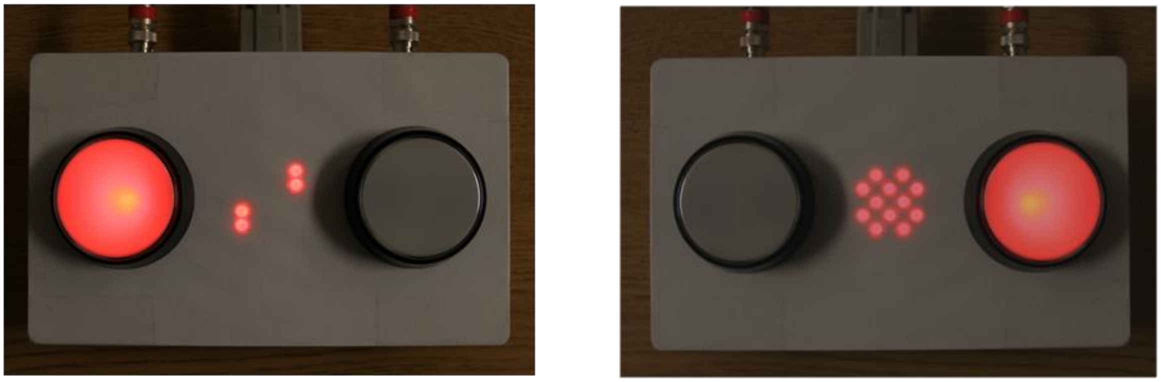

C.

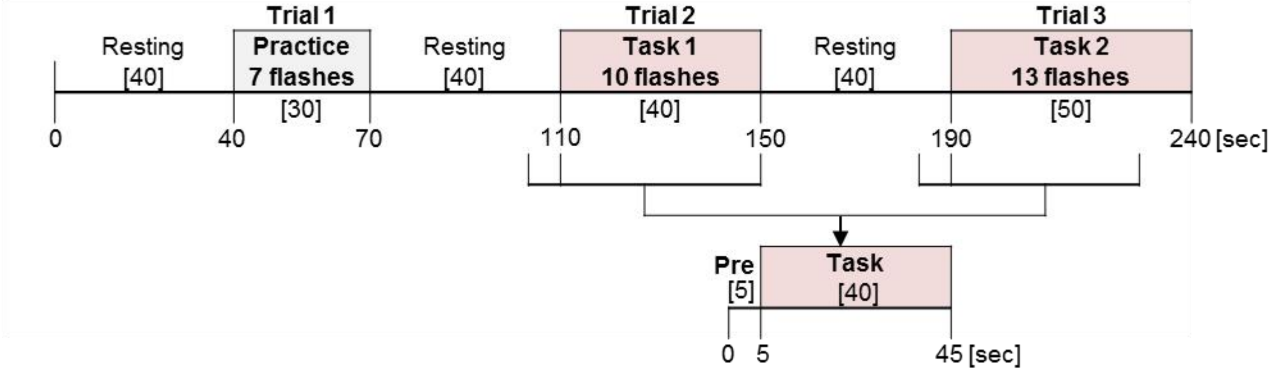

Resting
[40]
Trial 1
Practice
7 flashes
[30]
Resting
[40]
Trial 2
Task 1
10 flashes
[40]
Resting
[40]
Trial 3
Task 2
13 flashes
[50]
0
40
70
110
150
190
240 [sec]
Pre
[5]
Task
[40]
0
5
45 [sec]

D.

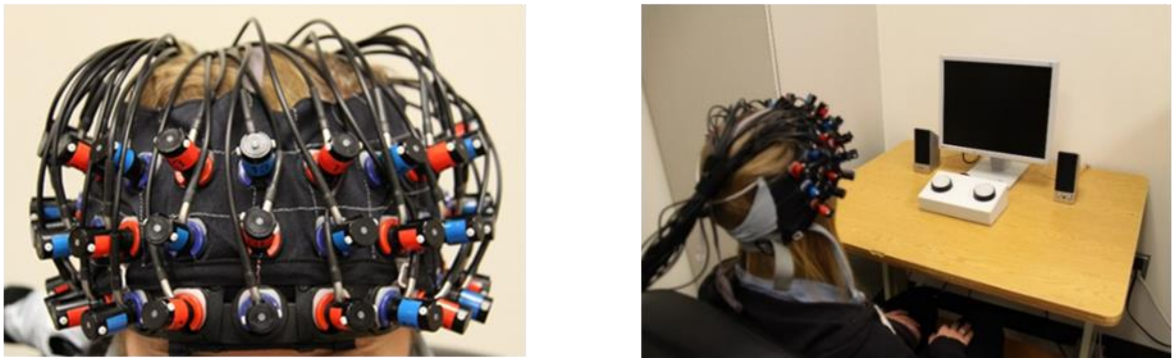

Figure Supplementary

Fig. 4. Near infrared spectroscopy (NIRS) with verbal fluency task and sustained attention task: (A) Task design of verbal fluency task, (B) Edinburgh Delirium Test Box Mark 2, (C) task design of sustained attention task, and (D) NIRS set up with Edinburgh Delirium Test Box Mark 2 and the display monitor for verbal fluency task.

Supplementary Table 1 Demographic and illness severity characteristics of patients with End Stage Liver Disease

Characteristic	Patients with End Stage Liver Disease					p value[b]
	Delirium[a]		No Delirium[a]			
**N**	12		46			
Medication (SD)						
Antidepressants, Imipramine-equivalent mg	51.1 (73.7)		42.3 (104.8)			0.794
West Haven Encephalopathy Criteria[c]	Grade 0	Grade I	Grade II	Grade III	Grade IV	< 0.001
(Delirium / No Delirium)	(0 / 11)	(1 / 18)	(6 / 17)	(5 / 0)	-	

[a] As measured using the Confusion Assessment Method (CAM)

[b] Significant group differences are shown to the right. *t* tests were used for testing group differences between delirium and no delirium in anti-depressants and benzodiazepine. Chi-squared test were used for testing grade differences between delirium and no delirium in West Haven Encephalopathy Criteria.

[c] Severity of hepatic encephalopathy (Grade 0: No impairment, Grade I: Minor cognitive impairment, Grade II: Moderate cognitive impairment, Grade III: Severe cognitive impairment, Grade IV: Coma)

Supplementary Table 2 Correlation coefficient for each channel between [oxy-Hb] integral value and DRS-R98 severity score in participant with end stage liver disease.

	Verbal fluency task		Sustained attention task	
channels	*r* value	*p* value	*r* value	*p* value
1	-0.163	0.304	0.389	0.011
2	-0.238	0.125	0.287	0.062
3	-0.201	0.196	0.086	0.581
4	0.080	0.605	0.256	0.102
5	0.017	0.914	0.259	0.093
6	0.078	0.618	0.450	0.002
7	0.061	0.692	0.408	0.005
8	-0.230	0.134	0.344	0.022
9	-0.036	0.821	0.430	0.004
10	-0.035	0.821	0.385	0.011
11	-0.186	0.244	0.141	0.374
12	-0.131	0.391	0.224	0.145
13	-0.027	0.866	0.219	0.153
14	-0.072	0.645	0.246	0.108
15	-0.200	0.198	0.153	0.321
16	-0.049	0.756	0.252	0.108
17	0.064	0.683	0.313	0.036
18	-0.063	0.680	0.527	< 0.001
19	-0.216	0.194	0.320	0.034
20	0.041	0.798	0.241	0.129
21	-0.028	0.860	0.433	0.003
22	-0.135	0.384	0.210	0.193
23	0.142	0.352	0.234	0.135
24	0.121	0.429	0.364	0.017
25	0.049	0.754	0.193	0.203
26	-0.169	0.268	0.160	0.293
27	0.046	0.766	0.290	0.054
28	-0.107	0.484	0.280	0.062
29	-0.139	0.392	0.600	< 0.001
30	0.176	0.303	0.195	0.211
31	-0.102	0.524	0.288	0.058
32	-0.002	0.988	0.180	0.248
33	-0.167	0.316	0.262	0.097
34	-0.052	0.742	0.257	0.096
35	0.000	0.998	0.220	0.147
36	-0.054	0.732	0.297	0.051
37	0.147	0.378	0.121	0.433
38	0.129	0.406	0.288	0.058
39	-0.272	0.086	0.477	0.001
40	0.112	0.492	0.332	0.034
41	-0.055	0.740	-0.022	0.894
42	-0.094	0.563	0.261	0.095
43	0.027	0.866	0.239	0.123
44	-0.245	0.128	0.179	0.252
45	-0.265	0.083	0.181	0.240
46	-0.329	0.027	0.239	0.114
47	-0.157	0.347	0.257	0.097
48	0.157	0.333	0.271	0.083
49	-0.119	0.458	0.274	0.068
50	-0.302	0.049	0.462	0.002
51	0.148	0.367	0.362	0.028
52	-0.107	0.505	0.267	0.101

Supplementary Table 3. Three groups comparison of integral value during Verbal Fluency Task

	Integral value [10-70 second]			Post hoc[e]		
	Delirium[d] (N=7)	No delirium[c] (N=38)	HC[b] (N=28)	Delirium > No delirium	HC > Delirium	HC > No delirium
Channel number[a]	mMmm (SD)	mMmm (SD)	mMmm (SD)	*p* value	*p* value	*p* value
Right postcentral gyrus						
Channel 2	10.0 (43.8)	10.4 (48.0)	48.2 (52.8)	n.s.	n.s.	0.011
Channel 13	33.5 (31.3)	14.8 (54.0)	62.4 (47.5)	n.s.	n.s.	0.002
Right precentral gyrus						
Channel 23	52.9 (31.6)	26.3 (60.6)	84.9 (83.3)	n.s.	n.s.	0.005
Right middle frontal gyrus					n.s.	
Channel 14	20.6 (38.6)	13.6 (45.9)	53.8 (55.5)	n.s.	n.s.	0.005
Channel 46	-21.3 (78.8)	22.0 (71.3)	117.1 (144.6)	n.s.	0.008	0.002
Right inferior frontal gyrus					n.s.	
Channel 24	50.7 (54.4)	15.9 (51.7)	73.3 (59.2)	n.s.	n.s.	< 0.001
Channel 35	29.0 (47.5)	15.6 (51.2)	85.0 (98.8)	n.s.	n.s.	0.001
Channel 45	11.4 (129.8)	42.0 (53.3)	108.0 (119.7)	n.s.	n.s.	0.016
Left precentral gyrus						
Channel 20	20.4 (38.6)	13.2 (65.1)	89.1 (69.0)	n.s.	0.035	< 0.001
Channel 30	55.2 (31.6)	24.8 (78.0)	113.8 (77.3)	n.s.	n.s.	< 0.001
Channel 31	37.0 (62.2)	24.8 (78.0)	99.7 (78.0)	n.s.	n.s.	0.005
Left postcentral gyrus						
Channel 9	10.1 (61.9)	13.2 (65.1)	65.1 (63.3)	n.s.	n.s.	0.006
Left middle temporal gyrus						
Channel 42	23.6 (43.9)	34.2 (90.5)	111.6 (83.7)	n.s.	0.045	0.002
Left inferior frontal gyrus						
Channel 40	70.0 (48.3)	32.4 (60.6)	110.0 (76.2)	n.s.	0.340	< 0.001
Channel 50	-0.75 (65.3)	58.5 (83.2)	116.1 (107.7)	n.s.	0.018	0.041

[a] Channels with significant differences among 3 groups by one-way analysis of variance (the false discovery rate corrected p value < 0.05)

[b] Healthy controls

[c] Patients with end stage liver disease (ESLD) with delirium as measured using the Confusion Assessment Method (CAM)

[d] ESLD with no delirium as measured using CAM

[e] Post hoc Tukey–Welsch tests were performed on these significant channels.
